# Polytrauma impairs fracture healing accompanied by increased persistence of innate inflammatory stimuli and reduced adaptive response

**DOI:** 10.1002/jor.26015

**Published:** 2024-11-17

**Authors:** Augustine Mark Saiz, Maryam Rahmati, Robert Charles Henry Gresham, Tony Daniel Baldini, Jane Burgan, Mark A. Lee, Benjamin Osipov, Blaine A. Christiansen, Thaqif El Khassawna, D. C. Florian Wieland, André Lopes Marinho, Clement Blanchet, Molly Czachor, Zachary M. Working, Chelsea S. Bahney, J. Kent Leach

**Affiliations:** ^1^ Department of Orthopaedic Surgery UC Davis Health Sacramento California USA; ^2^ California Northstate University College of Medicine Sacramento California USA; ^3^ Stony Brook Renaissance School of Medicine Stony Brook New York USA; ^4^ Experimental Trauma Surgery Justus‐Liebig University Giessen Giessen Germany; ^5^ Faculty of Health Sciences University of Applied Sciences Giessen Germany; ^6^ Institute of Metallic Biomaterials, Helmholtz Zentrum Hereon Geesthacht Germany; ^7^ European Molecular Biology Laboratory EMBL Hamburg Germany; ^8^ Steadman Phillippon Research Institute Vail Colorado USA; ^9^ Oregon Health Sciences University Portland Oregon USA; ^10^ University of California San Francisco California USA

**Keywords:** bone, fracture healing, immune response, nonunion, polytrauma

## Abstract

The field of bone regeneration has primarily focused on investigating fracture healing and nonunion in isolated musculoskeletal injuries. Compared to isolated fractures, which frequently heal well, fractures in patients with multiple bodily injuries (polytrauma) may exhibit impaired healing. While some papers have reported the overall cytokine response to polytrauma conditions, significant gaps in our understanding remain in how fractures heal differently in polytrauma patients. We aimed to characterize fracture healing and the temporal local and systemic immune responses to polytrauma in a murine model of polytrauma composed of a femur fracture combined with isolated chest trauma. We collected serum, bone marrow from the uninjured limb, femur fracture tissue, and lung tissue over 3 weeks to study the local and systemic immune responses and cytokine expression after injury. Immune cell distribution was assessed by flow cytometry. Fracture healing was characterized using microcomputed tomography (microCT), histological staining, immunohistochemistry, mechanical testing, and small angle X‐ray scattering. We detected more innate immune cells in the polytrauma group, both locally at the fracture site and systemically, compared to other groups. The percentage of B and T cells was dramatically reduced in the polytrauma group 6 h after injury and remained low throughout the study duration. Fracture healing in the polytrauma group was impaired, evidenced by the formation of a poorly mineralized and dysregulated fracture callus. Our data confirm the early, dysregulated inflammatory state in polytrauma that correlates with disorganized and impaired fracture healing.

## 1 INTRODUCTION

Trauma remains one of the leading causes of morbidity and mortality, and fractures are the major physical consequence of injury worldwide.^1^, ^2^, ^3^ Fracture healing is a multifaceted process that is dependent upon the activation of the immune system immediately after bone injury. This leads to the activation of local inflammatory cells and cytokines that, under normal conditions, stimulate the inflammatory, repair and remodeling phases of fracture healing.^4^ While it is well known that the loss of a proper, sequential immune response plays a role in impaired and disorganized bone healing,^5^, ^6^ this knowledge has been largely restricted to isolated fractures.

Polytrauma patients are typically defined by possessing an Injury Severity Score (ISS) of >15 and comprise a combination of multiple severe injuries such as bone fractures, blunt chest trauma, hemorrhage, head injury, abdominal visceral injuries, etc.^7^ Polytrauma results in a systemically dysregulated immune response.^5^ The severe imbalanced inflammation and immune response to multiple injuries may cause isolated or multiple organ dysfunction,^8^ which could negatively impact fracture healing.^9^, ^10^, ^11^ The incidence of failed healing (nonunion) is substantially higher in polytrauma patients compared to those patients with an isolated fracture.^12^ Furthermore, the number of inflammatory cytokines at the fracture site of immunosuppressed patients is dramatically altered compared to otherwise healthy patients, suggesting that local inflammatory changes could lead to delayed healing.^13^ Whether a similar mechanism occurs in polytrauma patients is unknown.

In the clinical setting, polytrauma results in a systemically dysregulated immune response.^5^ The damage‐associated molecular patterns and pathogen‐associated molecular patterns resulting from multiple injuries can cause isolated or multiple organ dysfunction,^8^ which amy negatively impact fracture healing.^9^ However, whether this systemic immune dysregulation affects the local fracture healing environment is unknown.

Although few studies have addressed the possibility of delayed fracture healing in patients with multiple fractures and body system injuries,^14^, ^15^ how local and systemic immune responses impact the fracture healing process in polytrauma remains unclear. Several studies have investigated the cytokine expression in different polytrauma animal models. For example, Fitschen‐Oestern et al.^16^ developed a standardized polytrauma mouse model with a femur fracture and blunt chest trauma to investigate the immune response to traumatic injuries that include bone fractures by mainly measuring the cytokine levels. Their results showed that the expression of interleukin‐6 (IL‐6) increases significantly 12 h after polytrauma.^16^ Relij et al.^17^ later compared the inflammatory response and organ damage of five isolated trauma with three multiple trauma models including healthy control, sham, hemorrhagic shock, thoracic trauma, osteotomy with external fixation, bilateral soft tissue trauma or laparotomy; polytrauma I; polytrauma II, and multi‐trauma group.^17^ Their cytokine analysis indicated that the systemic IL‐6 elevated in all mono and multiple trauma groups, whereas CXCL1 increased only multiple injured groups versus control.^17^ However, more studies are required to investigate the fracture healing and immune response to polytrauma in detail. Therefore, we investigated the local fracture site and systemic alterations of the innate and adaptive immune cell populations in the polytrauma mouse model developed by Fitschen‐Oestern et al.^16^ We hypothesized that an early dysregulation of the local fracture site immune response would lead to impaired fracture healing in the polytrauma models when compared to isolated injury models.

## 2 MATERIALS AND METHODS

### 2.1 Animal surgery

We performed animal surgeries in the Department of Orthopaedic Surgery, University of California, Davis in compliance with the ARRIVE guidelines under an approved protocol from the UC Davis Institutional Animal Care and Use Committee (IACUC 23193). Using bone formation at a single fracture site as our primary outcome variable, and based on our prior preliminary studies on bone formation,^3^ we quantified a mean difference of 42% between fracture and control groups. Based on these data and assuming equal standard deviations, we determined that eight animals per group would be necessary to provide a statistical power of at least 85% and detect a difference in bone formation rates due to polytrauma and bone fracture. Therefore, we acquired 208 10‐week‐old male C57BL/6 J mice (Jackson Laboratories). Mice were randomly housed in groups of four and were acclimated to the housing vivarium for 2 weeks before any procedures. Water and pellet diet were provided ad libitum. At 12 weeks of age, animals were randomly divided into four groups: healthy non‐injured, isolated blunt femur fracture (iso fracture), isolated blunt chest trauma (chest trauma), and polytrauma (femur fracture + chest trauma).

Mice were injected subcutaneously with buprenorphine (0.1 mg/kg) for pain control and saline 5–10 min before surgery. For inducing the femur fracture, mice were anesthetized with 2–4% isoflurane in oxygen and the right hind‐limb was shaved and prepped in a standard sterile manner. An incision was made over the anterior knee joint, and the distal femur was exposed. The knee was flexed, and the right femur was reamed through the femoral condyles with a 30 G needle as an intramedullary pin (IM). The joint capsule was closed using Monocryl (Johnson & Johnson, Brunswick, NJ) sutures and the skin closed with nylon sutures. We induced a transverse femur fracture by dropping a 30 g blunt weight on the diaphysis of the mouse femur, causing transverse bone fracture using an Einhorn device (Supporting information: Figure S1A). X‐ray imaging confirmed the correct placement of the needle and the induced fracture. The fracture apparatus has been described elsewhere.^15^, ^18^

After fracture and while still under anesthesia, we induced blunt thoracic trauma by dropping a hollow aluminum cylindrical weight (~30 g) from a height of 55 cm through a vertical stainless‐steel tube onto a Lexon platform resting on the animal's chest (Supporting information: Figure S1A). This apparatus has been described elsewhere.^16^ Mice were allowed to recover from anesthesia in a well‐ventilated area and kept warm using a heating pad underneath half of the cage with a surgical towel to minimize risk of contact thermal injury. Animals were observed for 10 days after surgery. Mice were injected with buprenorphine (0.1 mg/kg) twice daily after surgery (approximately 12 h apart) for 48 h. Mice were also weighed (for a minimum of 7 days) to ensure that no more than 20% of weight was lost from baseline weight. Baseline weight was the weight on the day of surgery, before surgery. This weight was used to calculate preoperative analgesia. The surgical site was examined daily until the sutures were removed or the animals were euthanized. The animals were full weight‐bearing and unrestricted activity was permitted postoperatively.

To account for temporal changes in the immune response, we considered both early and late time points (0, 6, 12, 24, and 72 h, and 3 weeks) in our study. Several studies have shown that 72 h is a proper last time point for the early phases of inflammatory responses to severe traumatic injuries.^19^, ^20^ Mice were euthanized by exsanguination via cardiac puncture under anesthesia followed by cervical dislocation. Blood was collected during cardiac puncture to analyze cytokines within the serum. Lung and femur tissues were dissected for downstream analyses.

### 2.2 Flow cytometry

To study the local and systemic immune response as well as the cellular phenotype of the immune system, we collected bone marrow from uninjured femur and tibia (by flushing it using a 25‐gauge needle), femur tissue from fractured femur and lung tissue after 0, 6, 12, 24, 72 h and 3 weeks from all mice (a total of 144 animals, 6 animals per time point per group). Since we collected approximately one million cells per tissue per mouse for flow cytometry, a total of 6 mice per group per time point was sufficient to provide statistical power of at least 85% and detect any potential differences between groups. Femur tissue was harvested by removing muscle around the tissue and cutting the bone from both the knee and hip sides. To preserve the periosteum, any remaining muscle was scrubbed off the femur with care. Femur was then cut into 1 mm pieces, minced, and filtered through 70 and 40 µm cell strainers to obtain the cell suspension. To harvest lung tissue, after exposing the thoracic wall muscles and the abdominal organs, we punctured the diaphragm and cut the ribs to expose the thoracic cavity. Through a small opening in the left ventricle and using a 27‐gauge needle, we infused the lung three times through the right ventricle with 8 mL PBS per infusion. Then, the lungs were harvested, cut into 1 mm pieces, minced, and filtered through 70 and 40 µm cell strainers to obtain the cell suspension.

Fresh bone and lung specimens were mechanically digested to small pieces and then enzymatically digested with collagenase VIII (0.5 mg/mL) (Sigma Aldrich, St. Louis, MO) and collagenase/hyaluronidase (Stem Cell Technologies) in RPMI 1640 medium (Gibco), respectively, for 45 min at 37°C. The digested tissues and bone marrow were processed through 70 and 40 μm cell strainers (Thermo Fisher Scientific), rinsed with PBS + 0.05% BSA, and then washed twice with 1× PBS. We washed and stained the enriched single‐cell suspension according to Motz et al.^21^ Briefly, we stained the cells using the fixable viability dye (Life Technologies, 65086514), CD45 (BioLegend, 103139), CD19 (BioLegend, 115554), CD3 (BioLegend, 100205), CD4 (BioLegend, 116014), CD8 (BioLegend, 344733), CD11b (BioLegend, 301356), Lys6G (BioLegend, 127612), F4/80 (BioLegend, 123114), and CD11c (BioLegend, 337210), and assessed our stained cells on a BD Fortessa 18‐color flow cytometer at the Institute of Regenerative Medicine, University of California, Davis. Data analysis was performed in FlowJo flow cytometry analysis software (©FlowJo, LLC 2024 | FlowJo v10).^21^

### 2.3 Analysis of cytokines within serum

Whole blood collected from cardiac puncture from the same mice that were used for flow cytometry. The sample preparation was performed according to the company protocol (Catalog#: K15048D‐1, Meso Scale Discovery, Rockville, MD).

### 2.4 3D microcomputed tomography (µCT) analysis

We imaged femurs (fixed in 4% paraformaldehyde (PFA)) from the healthy, isolated fracture and polytrauma groups (8 mice per group per time point, a total of 24 mice) using a specimen micro‐CT scanner (μCT 35, Scanco Medical; Wangen‐Brüttisellen, Switzerland). Scan parameters were 55 kVp, 145 μA, 300 ms exposure time, average of 3 exposures per projection, 0.5 mm aluminum filter, 500 projections per 180 degrees and a 10 μm nominal voxel size. The raw images were calibrated using a hydroxyapatite (HA) phantom of varying HA concentrations. Noise in the images was reduced by the use of a low‐pass Gaussian filter. A region of interest (ROI) was contoured to the outer edge of bone from the prominence of the lesser trochanter proximally to just proximal to the physes distally. Fabella and patella were excluded from the region. Each bone had a slightly different length, which resulted in analyzing different number of slices, because the ROI was based on anatomy. The mean+/−standard deviation of voxels/slices was 1816+/−135. Given the resolution was 0.01 mm in all dimensions, this means the length analyzed was 18.16+/−1.35 mm. We used a threshold of 470 mgHA/cc to partition mineralized tissue from other less dense tissues and a threshold of 1500 mgHA/cc to exclude higher dense metal and metal artifact. Bone volume fraction (BV/TV) was determined by dividing the number of voxels denser than the low threshold representing mineralized tissue (BV: bone volume) by the total number of pixels in the region (TV: total volume). The mean density of all material in the volume is apparent bone mineral density (BMD).

### 2.5 Quantification of mechanical properties in bone

To determine mechanical properties of healing bone, we performed torsional testing on fractured femurs in isolated fracture versus polytrauma groups using a material testing system (ElectroForce 3200, TA Instruments, New Castle, DE). Rotation was applied at a rate of 1 degree/sec until failure. The resulting torque and rotation data were recorded at 50 Hz and analyzed to determine callus stiffness, yield torque, ultimate torque, energy to failure, and post‐yield rotation.

### 2.6 Histological, enzyme histochemical, and immunohistochemical analyses

After µCT imaging, we performed both calcified and decalcified histology on our samples (24 mice for calcified histology (the same mice used for µCT) and 24 mice for decalcified histology). We used Movat Pentachrome stain to image different components of the connective tissue: mineralized tissue appears bright yellow, cartilage appears as blue‐green, and non‐mineralized tissue appears bright red.^22^, ^23^ We used alkaline phosphatase (ALP) and tartrate resistant acid phosphatase (TRAP) enzyme histochemistry as known biological markers for osteoblast and osteoclast activities, respectively.^3^, ^23^

We performed immunohistochemistry using an Opal™ Polaris Kit (Akoya Biosciences, Marlborough, MA) and stained tissue sections using the following primary antibodies: CD31/PECAM‐1 Antibody (1:500 µl, AF3628, Novus Biologicals), Neutrophil antibody [7/4] (1:500 µl, ab53457‐100 mg, Abcam), F4/80 antibody (1:500 µl, ab300421‐100 ml, Abcam) according to the company protocol (Automation Multiplex IHC Kit, Ayoka).^21^

### 2.7 Quantitative histomorphometry analysis

Sections (except from TRAP and Sirius Red) were imaged at 20× (3.09 pixel/μm) magnification using a Leica microscopy system (Leica DM5500 photomicroscope equipped with a DFC7000 camera and operated by LASX software version 3.0, Leica Microsystem Ltd, Wetzlar, Germany). Fiji ImageJ was used for histomorphometry measurements of stained sections. ImageJ (version 1.51r; NIH, Maryland, USA) was used as a platform to run the program. The Trainable Weka Segmentation (TWS) was used as the base to create an optimized script to analyze tissue formation parameters such as mineralization, new bone and cartilage formation, vascularization, as well as macrophages and neutrophil percentages. Histomorphometry measurements were performed as previously reported.^24^ Regarding TRAP staining for osteoclasts, the number and length of positive stained cells with 2 or more nuclei were measured using ImageJ. A combination of a higher amount of these cells and larger length indicated a higher osteoclast activity. For osteoblast activity, the intensity of positive stained ALP samples (dark blue) was measured in Fiji ImageJ using TWS.

### 2.8 Small angle X‐ray scattering/X‐ray diffraction (SAXS/XRD) analysis

Technovit‐embedded bone sections with 70 µm thickness were used to study the collagen/hydroxyapatite (HAp) orientation and the size of hydroxyapatite plates using SAXS/XRD analysis. Due to source and cost limitations, SAXS/XRD was conducted on one randomly chosen sample per group in isolated fracture and polytrauma groups. This allowed us to investigate the whole fracture site in both groups as our region of interest. Sections were measured at the synchrotron beamline P12,^25^ Petra III, Deutsches Elektronen‐Synchrotron (DESY) Hamburg, operated by the European Molecular Biology Laboratory (EMBL).^26^ To assess the mean crystal thickness (T parameter), we applied the stack and card model developed by Gourrier et al.,^27^ adjusting it to the data. The T parameter serves as a measurement of HAp platelet size and indicates the thickness of the HAp platelets.^28^

### 2.9 Statistical analysis

The datasets were assessed for normality using a Kolmogorov‐Smirnov test. Normally distributed data were summarized using mean and standard deviation, while non‐normally distributed data were summarized using median and interquartile range. Two‐way ANOVA on ranks was performed when the normality test failed using the Kruskal–Wallis test. Otherwise, multi‐factor regression was performed. All analyses were performed in GraphPad Prism 8 (GraphPad Software Company, San Diego, CA). Significant differences were presented as *p < 0.05, **p < 0.01, ***p < 0.001 and ****p < 0.0001. Statistical analysis of **SAXS/XRD** was performed in MATLAB using an ANOVA test. Data with a *p*‐value less than 0.05 was considered significant.

## 3 RESULTS

### 3.1 Animal model

We used radiography to confirm the placement of the IM pin and femur fracture (Supporting information: Figure S1A). Immediately after radiography, we induced blunt chest trauma using a standard drop weight device, resulting in bilateral hemopneumothoraxes. To confirm the induced injury to the lungs, we compared the Hematoxylin & Eosin (H&E) stained lung tissues of healthy and polytrauma mice and observed substantially more erythrocytes in the polytrauma lung, indicating hemorrhage after the chest trauma in this group (Supporting information: Figure S1B).

### 3.2 Local versus systemic innate immune response to polytrauma

**Figure 1:**
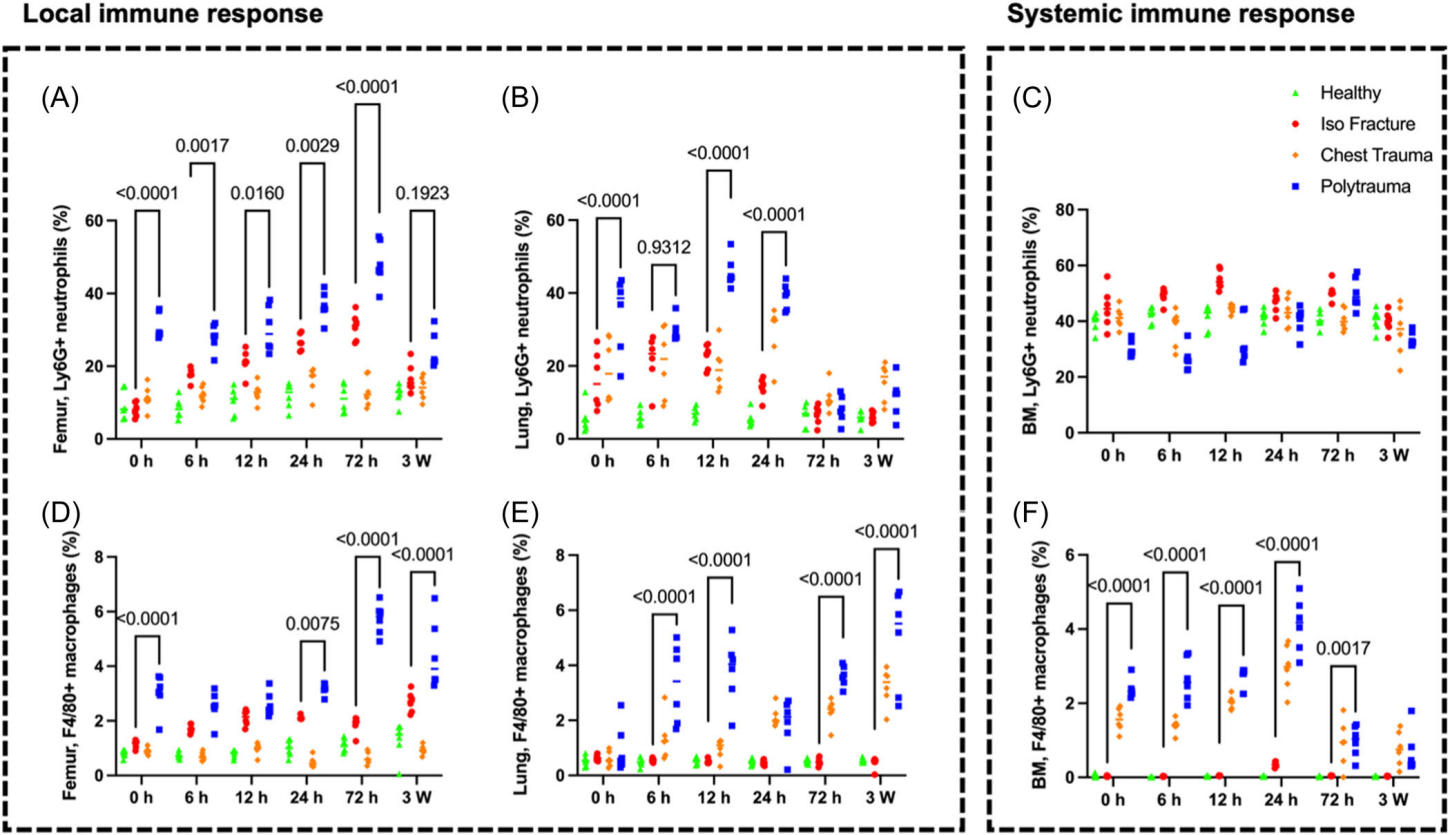
Polytrauma induces significant differences in local and systemic innate immune responses to fracture healing. Flow cytometry data comparing the percentage of neutrophils and macrophages at the femur fracture site (local, A and D), lungs (local, B and E), and bone marrow (systemic, C and F). BM (bone marrow). *N* = 6. Statistical comparisons are performed only between polytrauma and isolated fracture groups. The percentage of Ly6G^+^ Neutrophils and F480^+^ macrophages were calculated relative to CD45^+^ cells.

We studied the changes in the immune system after polytrauma and assessed both the innate and adaptive immune cell populations. The details of our gating strategy are depicted in Supporting information: Figure S2. Using flow cytometry, we characterized the percentage of Ly6G^+^ neutrophils and F4/80^+^ macrophages as the two major contributors of the innate immune system at the fracture site, lung tissue and bone marrow (BM) (Figure 1). We detected significantly higher percentages of neutrophils at the fracture site for the polytrauma group in the first 72 h compared to other groups (Figure 1A). Although a similar trend was observed in the lungs within the first 24 h of trauma (Figure 1B), the number of neutrophils in the lungs (Figure 1B) and bone marrow (Figure 1C) was comparable after 72 h of trauma in all experimental groups. The percentage of macrophages was higher in the polytrauma group compared to all other groups throughout the study duration at both fracture site and in the lungs after 0, 6, 12, 72 h and 3 w (Figure 1D,E). This was similar for the polytrauma group systemically (Figure 1F). However, this value dropped to the range of healthy mice after 3 weeks of injury.

A significantly higher expression of pro‐inflammatory cytokines such as IL6, and chemokine ligand 1 (CXCL1) was noted after 0 h and 3 weeks of trauma in the polytrauma group (Supporting information: Figure S3). The levels of IL‐10, an anti‐inflammatory cytokine, was lower for the polytrauma group compared to other groups with a significant decrease at 6 h. Regarding IL4, we did not observe any significant difference between groups (Supporting information: Figure S3).

**Figure 2:**
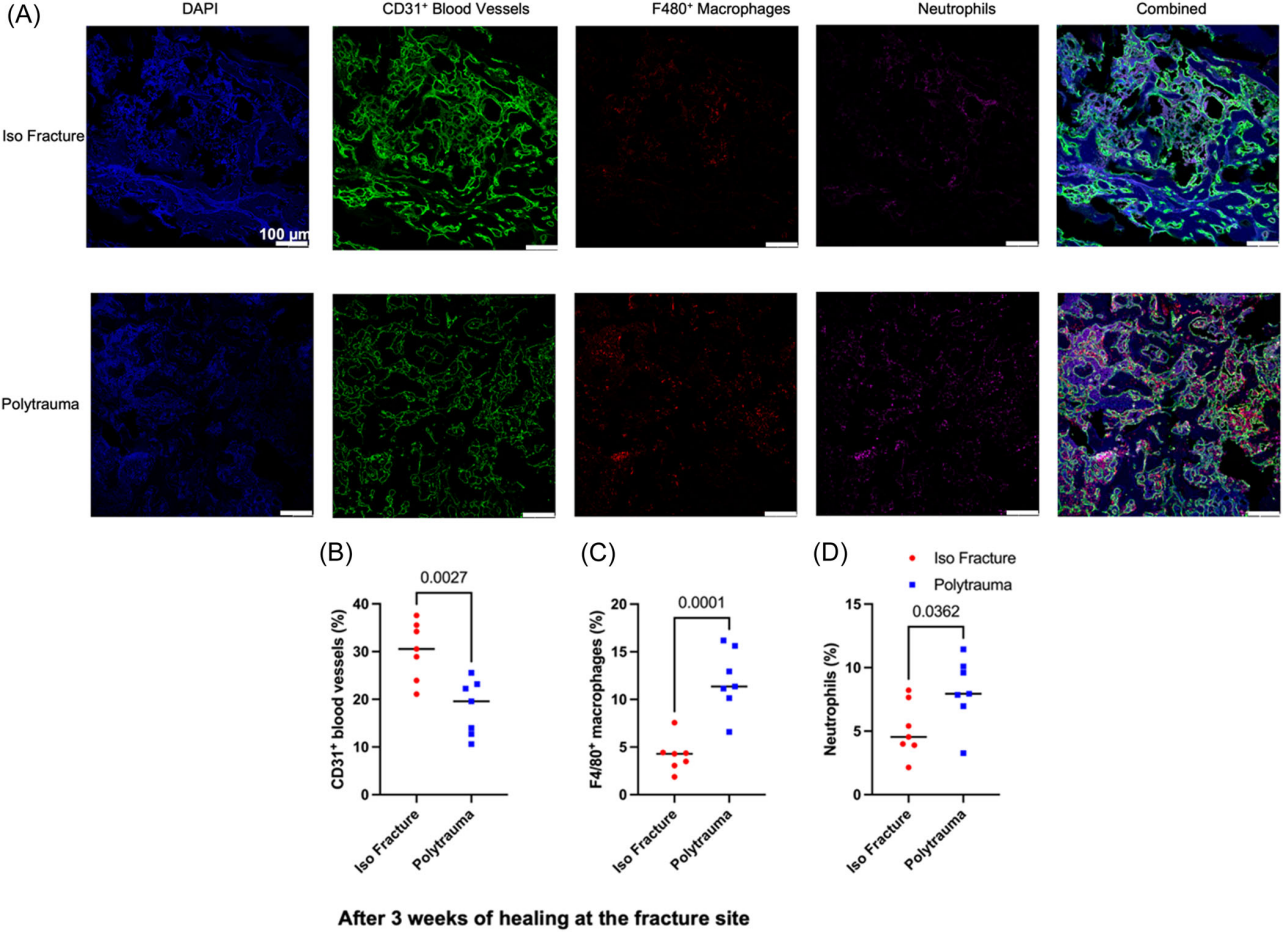
Polytrauma induces significant differences in local innate immune response to fracture healing. (A) Representative images of immunohistochemical analysis of CD31^+^ blood vessels (green), F4/80^+^ macrophages (red) and Abcam neutrophil antibody (magenta) at the fracture site (images shown were taken from the center of the fracture site) for isolated fracture and polytrauma groups after three weeks of healing. (B–D) Quantitative analysis of CD31^+^ blood vessels, F4/80^+^ macrophages, and neutrophils in percentages, respectively, compared between the two groups. Compared to our original power analysis (*N* = 8), the iso fracture (*N* = 6) and polytrauma groups (*N* = 7) experienced discrepancies in sample size due to sample loss during preparation or attrition from the study. Scale bar represents 100 µm (magnification 20x).

We performed multiplex immunohistochemistry to confirm our flow cytometry findings regarding the innate immune response to polytrauma after 3 weeks of healing on bone tissue at the fracture site (Figure 2A). The number of CD31^+^ blood vessels in the polytrauma group was lower (*p *= 0.0027) than in the isolated fracture group, indicating less angiogenesis (Figure 2B). The number of F4/80^+^ macrophages (*p *= 0.0001) and neutrophils (*p *= 0.0362) were higher in the polytrauma group than the isolated fracture (Figure 2C,D). These data confirmed our flow cytometry results shown in Figure 1 indicating that the mice with isolated fracture had immune and inflammatory resolution after 3 weeks of healing, whereas the polytrauma mice exhibit a persistent severe immune response to the trauma throughout the study timeline.

### 3.3 Local versus systemic adaptive immune response to polytrauma

**Figure 3:**
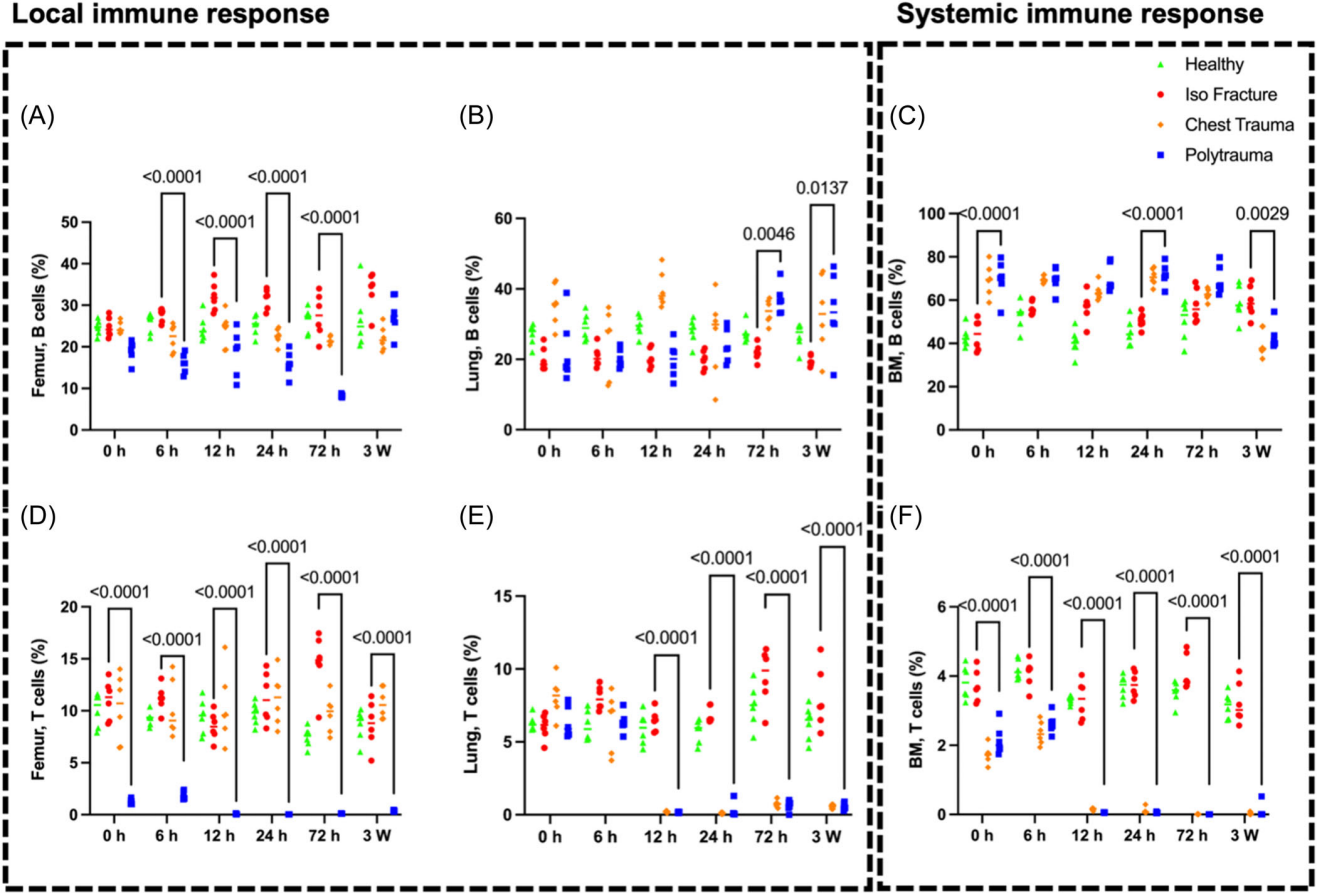
Polytrauma induces significant differences in local and systemic adaptive immune responses to fracture healing. Flow cytometry data comparing the percentage of B and T cells at the femur fracture site (local, A and D), lungs (local, B and E), and bone marrow (systemic, C and F). BM (bone marrow). *N* = 6. Statistical comparisons shown between the polytrauma and isolated fracture groups. The percentage of B and T cells were calculated relative to CD45^+^ cells.

Despite the higher percentage of innate immune cells in the polytrauma group, the frequency of adaptive immune cells was reduced in this group both locally and systemically (Figure 3). At the fracture site, the percentage of B cells was significantly lower in the polytrauma group than in other groups (Figure 3A). However, the number of bone marrow B cells was significantly higher in the polytrauma group after 24 h (Figure 3B). The number of bone marrow B cells decreased after 3 weeks in the polytrauma group (*p* = 0.0029) (Figure 3C). The presence of T cells was persistently reduced both locally (Figure 3D,E) and systemically (Figure 3F) in the polytrauma group (*p* < 0.0001) throughout the study duration.

Although the percentages of CD3^+^CD4^+^ helper and CD3^+^CD8^+^ cytotoxic T cells were significantly higher in the first 6 h of trauma in the polytrauma group, the percentage of these cells was reduced locally at the fracture site (Supporting information: Figure S4A,D) and in lungs (Supporting information: Figure S4B, E) (*p* < 0.0001 at 12, 24, 72 h and 3 weeks). Compared to the local fracture site and lungs, we only observed a significant difference between the isolated fracture and polytrauma group up to 72 h for CD3^+^CD4^+^ helper and CD3^+^CD8^+^ cytotoxic T cells, respectively, (Supporting information: Figure S4C,F).

### 3.4 Bone formation in polytrauma mice

**Figure 4:**
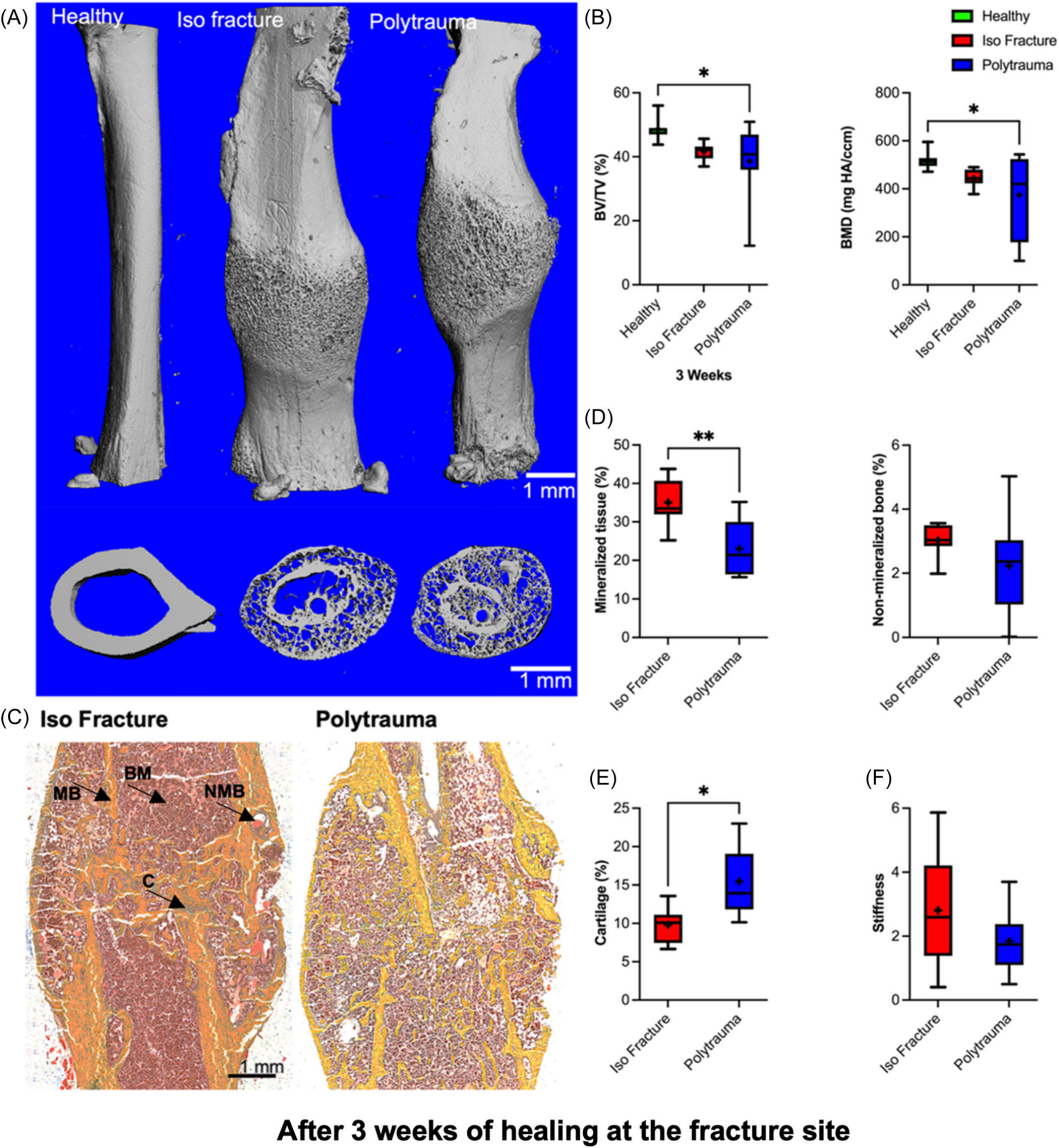
Polytrauma impairs fracture healing. (A) Representative microCT images of femur in healthy, isolated fracture, and polytrauma groups (C) and Movat Pentachrome histology for mineralized tissue (yellow, MB arrow), non‐mineralized tissue (red, NMB arrow), cartilage (green, C arrow), and bone marrow (brown, BM arrow) (scale bars = 1 mm) three weeks post‐injury. Quantitative analysis of (B) bone volume fraction (BV/TV) and bone mineral density (BMD); (D) mineralized and non‐mineralized tissue; (E) cartilage formation; and (F) and callus stiffness 6 weeks post‐injury (*N* = 8 for C–F). In F, compared to our original power analysis (*N* = 8), the iso fracture (*N* = 6) and polytrauma groups (*N* = 8) experienced discrepancies in sample size due to sample loss during preparation or attrition from the study. Significant differences between groups were presented with *p < 0.05 and **p < 0.001.

We examined the effect of polytrauma on bone formation and mineralization. Bone formation was analyzed using microCT. While we did not see any significant differences between polytrauma and isolated fracture groups regarding their BV/TV and BMD on microCT, (Figure 4A,B), fractures in the polytrauma group exhibited significantly lower bone volume fraction and bone mineral densities than the healthy baseline group after 3 weeks of healing (Figure 4A,B). Importantly, we observed a high degree of variability in bone formation after polytrauma that was not observed in the isolated fracture group. Using pentachrome staining, bone mineralization and fracture healing (yellow) were homogeneous in the isolated fracture group (Figure 4C). However, there was no fracture gap closure in most of the polytrauma samples with a lower percentage of bone mineralization noted (*p *= 0.003) (Figure 4D). We observed more cartilage tissue (green) (Figure 4E) in the polytrauma group (*p *= 0.0062). The torsion test revealed no significant differences in stiffness between the polytrauma and isolated fracture groups (Figure 4F & Supporting information: Figures 5A–C).

### 3.5 Bone turnover and maturity in polytrauma

**Figure 5:**
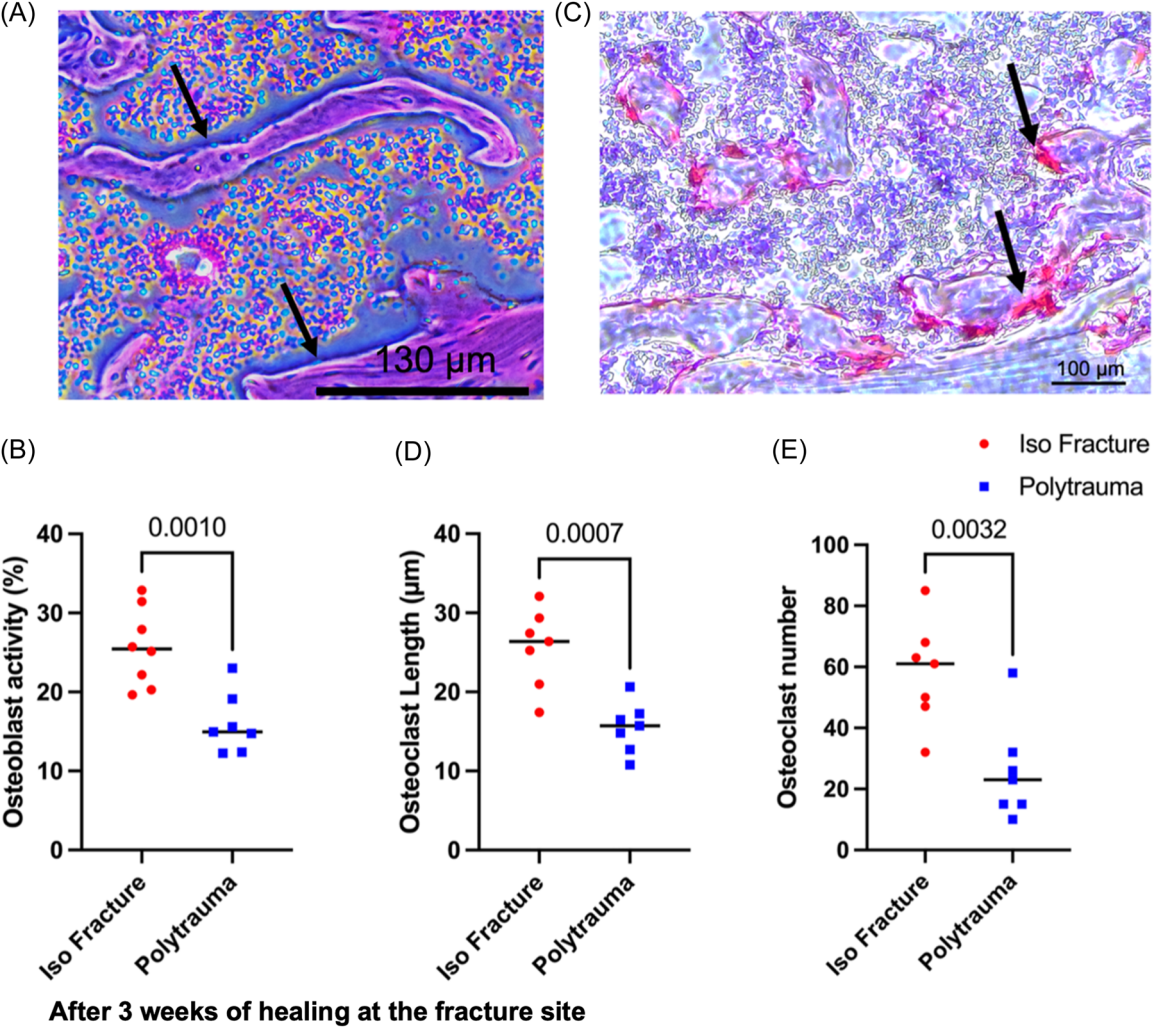
Polytrauma dysregulates bone turnover at the fracture site. Representative images of (A) alkaline phosphatase (ALP) (20x magnification, scale bar is 130 µm) and (C) tartrate‐resistant acid phosphatase (TRAP) staining (40x magnification, scale bar is 100 µm) to quantify the activity of osteoblasts (black arrows) (B) and osteoclasts (D&E) (black arrows), respectively, after three weeks of healing. Compared to our original power analysis (*N* = 8), the iso fracture (*N* = 7) and polytrauma groups (*N* = 7) experienced discrepancies in sample size due to sample loss during preparation or attrition from the study.

We subsequently studied bone formation and absorption rates by histological analysis by assessing ALP and TRAP enzyme activity (Figure 5). In decalcified samples, ALP positive area was lower in the polytrauma group than in the isolated fracture after 3 weeks (*p *= 0.01) (Figure 5A,B). The number of osteoclasts, ascertained via TRAP staining and quantifying cell length, was significantly decreased in the polytrauma group (Figure 5C–E). These data revealed that bone turnover rate is significantly lower in the polytrauma group.

**Figure 6:**
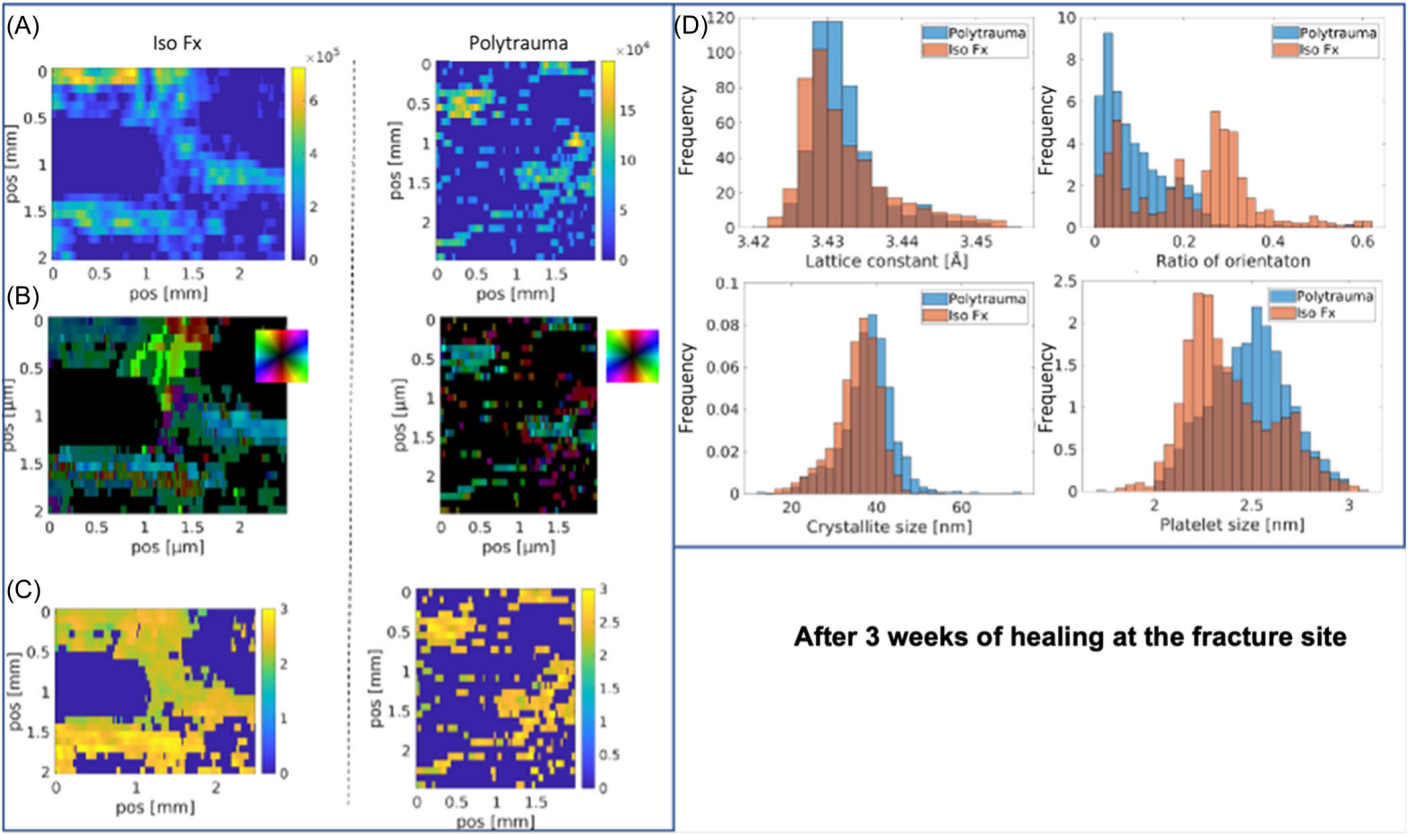
Polytrauma leads to less bone maturity after healing. Small angle X‐ray scattering/X‐ray diffraction (SAXS/XRD) analysis of the osteogenesis and biomineralization processes at the fracture site in isolated fracture vs polytrauma groups after three weeks of healing. (A) Two‐dimensional scattering maps. The strength of the scattered intensity directly correlates to the mass amount in the probed voxel. (B) Orientation of the HAP/collagen matrix. The orientation of the axis of the bone is horizontal. The color wheel directly shows the orientation as indicated in the top right corner, with red being an orientation in the vertical direction and cyan in the horizontal direction. The color brightness indicates the degree of orientation. (C) T parameter giving information about the HAp platelet size. (D) Histograms of the degree of orientation, lattice constant of the 002 reflection, T parameter, and the crystal size.

The bone matrix is a composite material consisting of collagen and hydroxyapatite (HAp) platelet parameters and orientation. The HAp in the mineralized tissue between the fibers and the growth is constrained by available space. Therefore, the HAp growth in the shape of platelets with the c‐axis of the crystal is parallel to the collagen fibers. Thus, the evaluation of the small angle scattering data allows the deduction of platelet size, which corresponds to the so‐called T parameter^27^ and the alignment of the HAp/collagen matrix as both are coaligned. Samples were cut along their long axis and the SAXS/XRD experiments were used to probe the variations across the induced fracture. Figure 6A–C & Supporting information: Figure S6 show the two‐dimensional intensity distribution of the SAXS signal, and the orientation of HAp. The orientation of the bone axis is in the horizontal direction. The isolated fracture group had much higher mineralization and better HAp alignment after 3 weeks, whereas no ordering was seen in the polytrauma group. We note that the color wheel directly shows the orientation as indicated in the top right corner, with red being an orientation in the vertical direction and cyan in the horizontal direction. The color brightness indicates the degree of orientation.

As a quantitative analysis, we calculated the median for the extracted values of the crystal size and lattice constant determined on the XRD data and the T parameter and degree of orientation extracted from the SAXS data (Figure 6D, Supporting information: Figure S7). The median of the lattice constant was 3.433 for both groups with no statistically significant variation. This was different for the crystal size and T parameter as the isolated fracture group showed a smaller platelet size (T parameter) and a smaller crystallite size. The degree of orientation of the isolated fracture was significantly higher as compared to the polytrauma group indicating a much better aligned collagen/HAp matrix. This observation suggests that the overall bone matrix for the isolated fracture group was already much better remodeled and mature compared to the polytrauma group. In a normal healing process, woven bone is formed first, which is later remodeled to cortical/trabecular bone. The HAp in this stage is typically much larger compared to a mature state and the collagen matrix is much less oriented/organized. Therefore, the data demonstrate a more mature bone structure in the fractured area of the isolated fracture group. These data align with our data obtained from bone turnover and histological analyses.

## 4 DISCUSSION

This study demonstrates that fracture healing is impaired in a polytrauma environment and proposes a dysregulated immunomodulatory etiology that occurs early and persists. Our findings agree with previous clinical studies performed on polytrauma patients.^10^, ^12^, ^29^ Here, we used a polytrauma mouse model with a combination of a blunt chest trauma and femur fracture. This mouse model reproduces the severe traumatic injuries observed in high‐energy trauma and the influence on fracture healing.^29^ Since each stage of the healing process can be susceptible to local and/systemic factors that disrupt the complex repair cascades, we studied the local and systemic immune responses to polytrauma as well as bone formation and maturity in our model. Our 3D µCT, 2D histological and immunohistochemical, and SAXS/XRD data confirmed that compared to isolated injuries, polytrauma mice exhibited significantly less angiogenesis, bone mineralization, bone metabolism rate and maturity. The induced transverse fracture is a non‐critically sized defect that is known to heal without any intervention.^30^ Previous in vivo studies demonstrated altered and delayed wound healing in polytrauma animals when compared to lesser forms of trauma.^10^, ^12^, ^29^ Magnum et al.^29^ observed a substantial reduction of bone volume fraction after 5 weeks of trauma in a noncritical‐sized defect with no signs of bone union in a polytrauma rat model of skin burn and femur fracture.^29^ In severe injuries or polytrauma cases such as our model, an imbalanced immune response can lead to acute and chronic complications such as isolated or multiple organ dysfunction and failures of healing such as infection or nonunion.^8^, ^31^

Flow cytometry, cytokine expression, and immunohistochemical analysis confirmed that the inflammatory and immune responses to polytrauma dramatically differed from isolated injuries. This confirmed our hypothesis regarding the negative impact of immune dysregulation on fracture healing in the polytrauma group. Most studies in the field have quantified changes in blood concentrations of cytokines and chemokines as an indicator of the immunologic response to trauma.^14^ Neunabar et al.^32^ studied the IL‐6, TNF‐α, CCL2, CCL3, CCL4, CCL5 and CCL7 cytokine expression in a similar polytrauma mouse model.^32^ They observed that compared to the isolated fracture, the inflammatory response was persistent in this polytrauma model after 3 days.^32^ While these studies contributed to identifying the temporal changes of these mediators, identifying the role and importance of each cytokine in predicting the immune resolution or suppression of polytrauma patients over longer time points is challenging.^14^ This could be due to the half‐life of individual cytokines, the time of its peak production, and the blood collection time point.^33^, ^34^ For instance, we could not successfully detect TNF‐α and IL‐1β in our collected serum, which could be because these cytokines are hyperacute pro‐inflammatory cytokines that are highly expressed within the first 2 h of polytrauma. Because of the short time of their peak production, these two markers cannot be considered as reliable predictors of immune suppression and multiple organ failure disorder development.^33^, ^34^ IL‐1β cytokine stimulates the endothelial cell adhesion, chemotaxis of PMNs and macrophages. In addition, its half‐life is approximately 10 min, making its clinical measurement difficult.^33^, ^34^ However, we were able to detect IL‐6, CXCL1, IL‐4 and IL‐10. We detected significantly higher IL‐6 and CXCL1 concentrations immediately after trauma and after 3 weeks of healing in the polytrauma group, while IL‐10 was lower at 6 h in polytrauma mice compared to those with isolated injuries. We did not detect differences in IL‐4 expression between groups. Our IL‐6 data were consistent with the results from Recknagel et al.^10^, ^11^ Compared to the isolated fracture, they observed significancy higher IL‐6 staining within the periosteal callus in zones of intramembranous ossification after 3 days of trauma^10^ and significantly higher IL‐6 after 6 and 24 h of injury in a polytrauma rat model.^11^

In addition, our findings are consistent with previous studies demonstrating that IL‐6 is highly active within the first 4 h and reaches a sustained level afterward, making this marker easier to measure than TNF‐α and IL‐1β.^35^, ^36^ Guisasola et al.^37^ did not observe any systemic pro‐inflammatory IL‐1β, TNF‐α or IL‐6 response to polytrauma.^37^ Therefore, limitations with cytokine half‐life as well as discrepancies between the existing data in the literature motivated us to focus on evaluating the changes in immune cell populations in greater depth using flow cytometry and immunohistochemistry.

We observed significantly greater percentages of neutrophils and macrophages in the polytrauma group compared to other groups. Although the percentage of these cells decreased systemically after 72 h, levels remained dramatically high at the fracture site. This is consistent with the conclusions from Bastian et al.^4^ demonstrating a higher neutrophil count in the blood. Additionally, neutrophil priming leads to greater neutrophil infiltration into the fracture site, hampering downstream healing.^4^ In polytrauma patients, neutrophils become more resistant to apoptotic processes^38^ and therefore not only attack injured cells and microorganism but also remaining healthy host cells resulting in the “second hit”.^39^, ^40^ Raghavendran et al.^41^ studied the inflammatory effects of lung injury in a bilateral lung contusion model in rats.^41^ They reported that the levels of erythrocytes, leukocytes, and albumin were increased at the early hours (less than 24 h), and returned toward normal by 48 h. In addition, the levels of macrophage inflammatory polypeptide‐2 (MIP‐2), cytokine‐induced neutrophil chemoattractant‐1 (CINC‐1), and IL‐6 were increased most at 24 h, whereas monocyte chemoattractant protein‐1 (MCP‐1) and IL‐1β were highest at 24 to 48 h post‐contusion. They also observed that inflammatory injury to lungs is most severe in the early hours after initial blunt trauma and has a component of neutrophil‐dependent pathology.^41^ In addition, the retrospective clinical study by Bastian et al.^12^ demonstrated that the myeloid cell counts are higher than normal in polytraumatic patients. They reported that leukocyte kinetics vary substantially between patients with normal and impaired fracture healing during the first 2 weeks of trauma, suggesting the potential role of systemic immune response on fracture healing. Mangum et al.^29^ examined the difference in innate and adaptive immune response in a rat polytrauma model consisting of a femur osteotomy, blunt chest trauma, and full‐thickness burn versus a single femur osteotomy.^29^ They found that polytrauma reduces the bone volume fraction and changes the concentration of cytokines. In the polytrauma group, macrophage infiltration was absent with no macrophages found at the fracture site 24 and 72 h post fracture. The pro‐inflammatory protein high mobility group box 1 (HMGB1) and the receptor for the macrophage inflammatory protein‐3 were significantly increased 24 h postfracture. Both pro‐ and anti‐inflammatory cytokines were significantly decreased at 24 and 72 h post fracture. Although these data did show an increase in pro‐inflammatory cytokines, the increase in pro‐inflammatory HMGB1 corresponds with other studies that found a high pro inflammatory early response followed by a systemic suppressed immune system in the polytrauma group.^13^, ^42^, ^43^ In a study comparing two isolated fractures (one with fracture and thoracic trauma, and the other with fracture and thoracic trauma plus another soft‐tissue trauma), IL‐6 levels were tripled after 1 day of fracture initiation, which was associated with decreased callus volume leading to impaired healing.^36^ Additionally, Recknagel et al.^10^ observed a significant depletion of macrophages in their polytrauma model after 3 and 7 days of trauma.^10^ In a rat open tibial fracture with extensive muscle volumetric loss model, there was a heightened CD68+ macrophage and lymphocyte response at the fracture callus 3 and 14 days postfracture.^44^ In a murine model of fracture and thoracic trauma with resulting comprised healing, Kovtun et al., depleted neutrophils using an anti‐Ly‐6G‐antibody, yet this did not improve fracture healing.^45^ These data suggest that neutrophils may not play a crucial pathological or mechanistic role in compromised fracture healing induced by an additional thoracic trauma. As such, continued investigation is required to understand how underlying immunologic mechanisms in polytrauma lead to impaired fracture healing.

In contrast, the percentage of adaptive B and T cells was significantly lower in the polytrauma group compared to other groups both systemically and locally. This difference was more dramatic for T cells and their CD3^+^CD4^+^ helper and CD3^+^CD8^+^ cytotoxic subtypes indicating the possibility of immunosuppression in this group. This could be related to immunotolerance and immunosuppression in severe injuries. Immune tolerance is a series of immunologic responses that deactivate the systemic immune syndrome that was originally initiated by systemic inflammatory response syndrome (SIRS) and a massive presence of neutrophils and macrophages. Although the immune tolerance decreases the severity of the SIRS pro‐inflammatory response, an imbalance between SIRS and immune tolerance could lead to morbidity and immunosuppression in polytrauma.^46^ In addition, these results were consistent with Debler et al.^47^ using a more severe mouse polytrauma model (traumatic brain injury, a closed transverse femoral fracture with soft tissue injury, and a blunt bilateral chest trauma with additional hemorrhagic shock).^47^ Their model showed upregulation of myeloid leukocyte activation and differentiation, upregulation of IL‐6, IL‐1β and TNF‐α, upregulation of genes involved neutrophil chemotaxis as well as a downregulation of pathways related to B‐ and T‐cell adaptive system.^47^

Based on our study and prior literature, the poor and dysregulated fracture healing that we observed in the polytrauma group originated from the imbalanced immune response. We believe that the continuous high percentage of neutrophils and macrophages at the fracture site led to impairing the activity of the adaptive immune system. This consequently hampered the bone mineralization through lower blood vessel formation, lower osteoblast and osteoclast activity, and decreased bone formation. It is conceivable that the reduced activity of osteoclasts in this group may have contributed to the delayed cartilage resorption in the polytrauma group. However, since we flushed BM out of callus for our flow cytometry analysis and the results from neutrophils and macrophages corroborated with our IHC results (where we did not remove BM), it is less likely that the increased number of immune cells seen after 3 weeks of healing is related to any changes in bone marrow volume at this time point.

A statistical limitation of our study is we did not account for the high variability in the polytrauma group when calculating our sample size. Our power analysis was based on prior results from isolated bone regeneration studies. Given the high variability and discrepancies in our findings, it is evident that a sample size of 8 is insufficient for detecting all differences in a polytrauma model. Future studies should account for this variability in their power calculations. This limitation could also be seen as an advantage of using a polytrauma model with fractures, as it more accurately reflects clinical outcomes compared to isolated fracture models. The high variability in response to polytrauma could be because of the tolerance/sensitivity level that each individual mouse has to the hemorrhage caused by chest trauma. McKinley et al.^48^ carried out a clinical study between polytrauma patients and observed that although two groups of patients had similar injury levels and demographic homogeneity, their sensitivity to hemorrhage was different. Tolerant cases had a high level of hemorrhage with insignificant organ dysfunction in contrast to sensitive cases who had less hemorrhage but significant organ dysfunction.^48^ In addition, compared to our original power analysis (*N* = 8), the iso fracture and polytrauma groups experienced discrepancies in sample size (e.g., Figures 2 and 5) due to sample loss during preparation or attrition from the study. Finally, microCT did not detect a statistically significant difference between polytrauma and isolated fracture groups in terms of BV/TV and BMD as opposed to pentachrome staining. There was a trend in the microCT and statistical significance may not have been reached given the difference in sample sizes or the heterogeneity in the setting of the fracture ROI.

## 5 CONCLUSION

Polytrauma is associated with increased risk of fracture nonunion in human patients. In our murine model of polytrauma with a stabilized femur fracture and thoracic blunt injury, we observed persistently altered innate and adaptive immune system responses both systemically and locally at the fracture site. This dysregulated immune response was associated with poorer fracture healing consisting of less organized fracture callus, decreased bony metabolism, and decreased mineralization at the fracture site. In polytrauma, there are a myriad of complex physiologic processes that likely affect fracture healing. Further research into the underlying mechanisms may improve understanding leading to therapeutic targets and enhanced treatment algorithms resulting in improved patient outcomes.

## AUTHOR CONTRIBUTIONS

**Augustine Mark Saiz**: Conceptualization; funding acquisition; investigation; methodology; project administration; resources; supervision; validation; writing—original draft; writing—review and editing. **Maryam Rahmati**: Conceptualization; investigation; methodology; writing—original draft; writing—review and editing. **Charles Henry Robert Gresham**: Data curation; formal analysis; investigation; methodology; validation. **Tony Daniel Baldini**: Investigation. **Jane Burgan**: Investigation. **Mark A. Lee**: Conceptualization; funding acquisition; methodology; project administration; resources; supervision. **Benjamin Osipov**: Investigation; visualization. **Blaine A. Christiansen**: Formal analysis; resources; writing—review and editing. **Molly Czachor**: Data curation; formal analysis. **Zachary M. Working**: Formal analysis; methodology. **Chelsea S. Bahney**: Conceptualization; formal analysis. **Thaqif El Khassawna**: Conceptualization; investigation; methodology; resources; validation; writing—review and editing. **D.C. Florian Wieland**: Investigation; methodology; software; writing—review and editing. **André Lopes Marinh**: Investigation; methodology; writing—review and editing. **Clement Blanchet**: Resources; writing—review and editing. **J. Kent Leach**: Conceptualization; methodology; project administration; resources; supervision; writing—review and editing. All authors have read and approved the final submitted manuscript.

## CONFLICT OF INTEREST STATEMENT

Augustine MarkSaiz serves on committees for OTA, AAOS, and ORS. The authors declared no potential conflicts of interest with respect to the research, authorship, and/or publication of this study.

## Supporting information

Supporting information.
