# Supplementary material for: Polytrauma impairs fracture healing accompanied by increased persistence of innate inflammatory stimuli and reduced adaptive response
Source: J Orthop Res. 2024 Nov 17;43(3):603–16. doi: 10.1002/jor.26015 (PMC11806648; doi:10.1002/jor.26015)
Supplement: Supplementary file 1 — Supporting information. [file JOR-43-603-s001.docx]

**Polytrauma impairs fracture healing accompanied by increased persistence of innate inflammatory stimuli and reduced adaptive response**

*Augustine Mark Saiz^1#^, Maryam Rahmati^1^*, Robert Charles Henry Gresham^1^*, Tony Daniel Baldini^1,2^, Jane Burgan^1,3^, Mark A. Lee^1^, Benjamin Osipov^1^, Blaine A. Christiansen^1^, Thaqif El Khassawna^4,5^, D.C. Florian Wieland ^6^, André Lopes Marinho^6^, Clement Blanchet^7^, Molly Czachor^8^, Zachary M. Working^10^, Chelsea S. Bahney^8,9^, J. Kent Leach^1^*

*^1^Department of Orthopaedic Surgery, UC Davis Health, 4860 Y Street, Suite 3800, Sacramento, CA, 95817, USA*

*^2^California Northstate University College of Medicine, CA, USA*

*^3^Stony Brook Renaissance School of Medicine, NY, USA*

*^4^Experimental Trauma Surgery, Justus-Liebig University Giessen, Giessen, Germany*

*^5^Faculty of Health Sciences, University of Applied Sciences, Giessen, Germany*

*^6^Institute of Metallic Biomaterials, Helmholtz Zentrum Hereon, Geesthacht, Germany*

*^7^European Molecular Biology Laboratory EMBL, Hamburg, Germany*

*^8^Steadman Phillippon Research Institute, Vail, CO, USA*

*^9^University of California, San Francisco, CA, USA*

*^10^Oregon Health Sciences University, Portland, OR, USA*

** These authors contributed equally to this study.*

*# Email of the Corresponding Author:* [*amsaiz@ucdavis.edu*](mailto:amsaiz@ucdavis.edu), Tel: [(916) 734-2700](tel:(916)%20734-2700), Fax number: [(916) 734-7137](tel:(916)%20734-7137)

**Running title**: Polytrauma and fracture healing


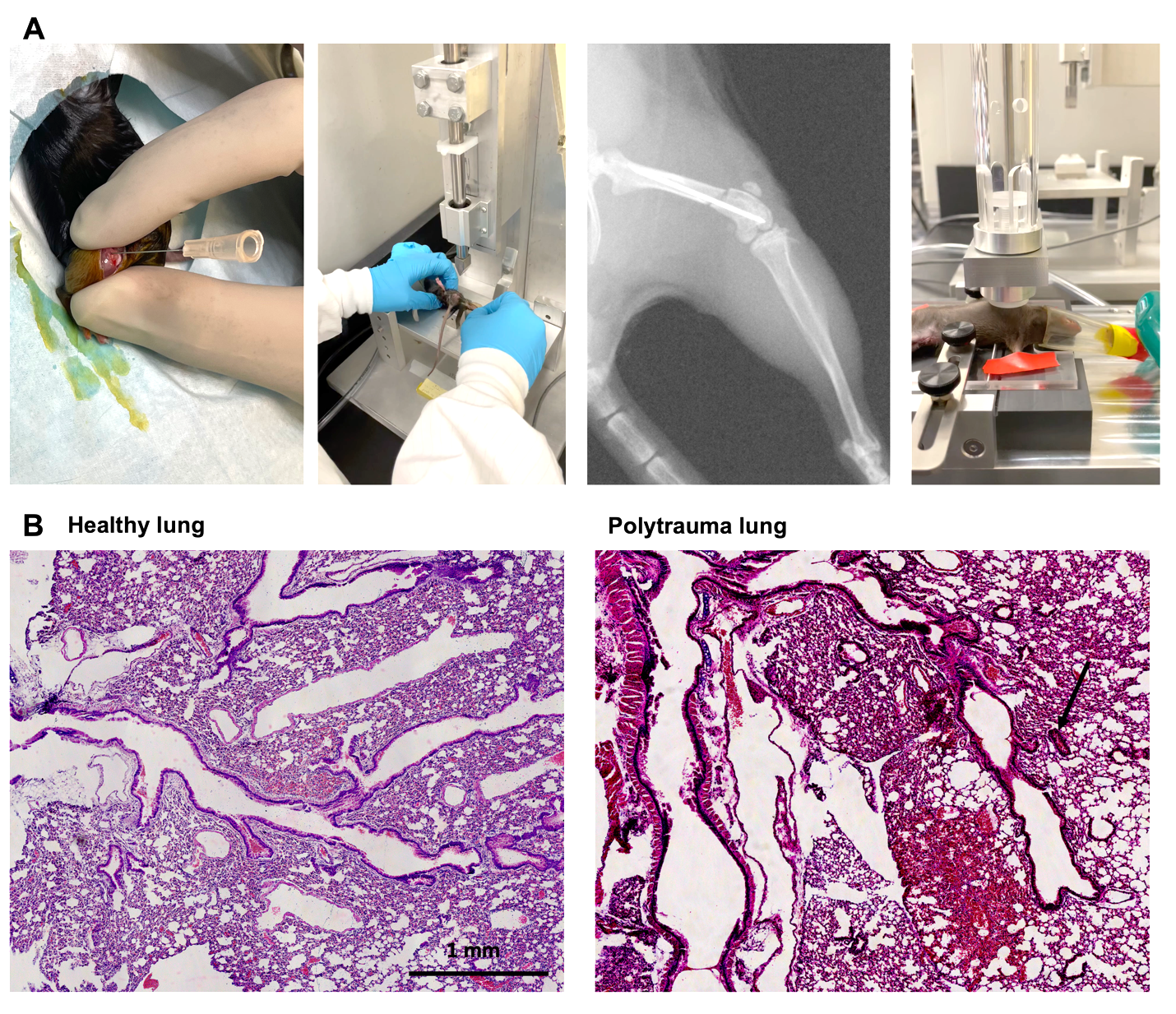


**Supplementary Figure 1.** Representative images of the polytrauma model (femur fracture + chest trauma). **A)** Representative images from left to right of inducing polytrauma through inserting a 30 G needle as our intramedullary pin (IM), inducing the blunt fracture using an Einhorn drop weight device, radiographical assessment of the defect site, and inducing the chest trauma using a standard drop weight device. **B)** Representative images of the Hematoxylin & Eosin (H&E) stained lung tissues from healthy and polytrauma mice, black arrow indicates erythrocytes. Scale bar = 1 mm.


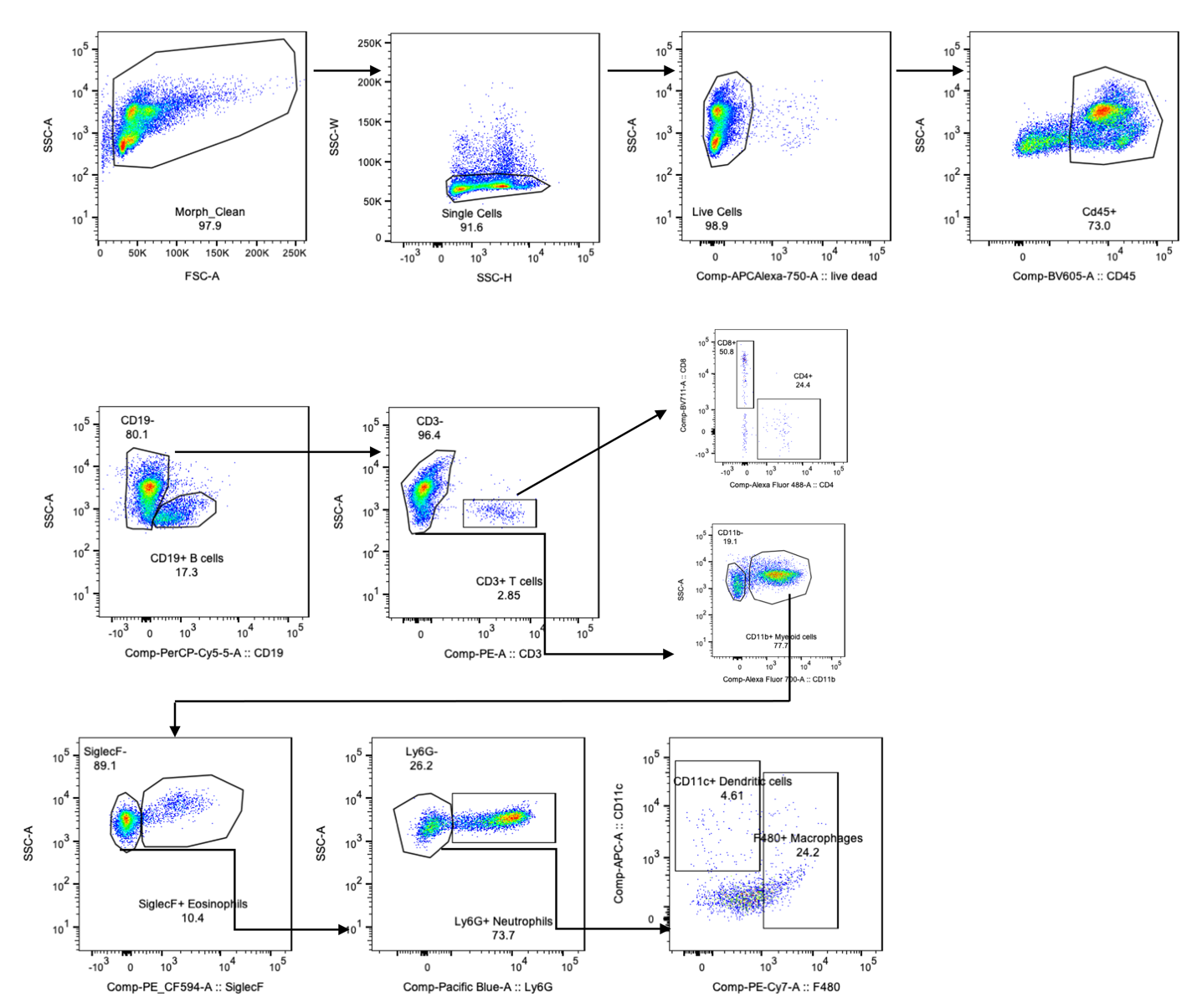


**Supplementary Figure 2.** Gating strategy for flow cytometry. Depicted is a representative image of the multicolor flow cytometry gating strategy used to determine the population of different immune cell population in single cell suspensions from femur, lungs, and bone marrow (BM). This representative image was made using one of the BM samples. However, a comparison between the gating strategy of all three tissues were made as a pilot study, and we did not see any significant difference regarding which tissue should be chosen as our standard method of gating. Since it was more likely to see immune cells in BM than other cells, we selected BM. Black contours show the positively stained populations of interest for each marker. After gating the debris using side and forward scatters (SSC and FSC) as our axes, we gated single cells from our non-debris (Morph-Clean) cells using the width and height of SCC. From our single cell population, we gated live cells using the Alexa 750-A live dead marker, which shows the live cells on the far-left side and the dead cells on the far-right side of the gate. From live cells, we gated CD45+ cells as our immune cell populations. From these cells, we gated CD19+ cells as our B cells and CD19- cells as our negative B cells. From CD19- cells, we gated CD3+ cells as our T cells and CD3- cells as our non-T cells. From CD3+ cells, we gated CD4+ and CD8+ T cells. From CD3- cells, we gated CD11b+ cells as myeloid cells and CD11b- as non-myeloids. From CD11b+ cells ls, we gated SiglecF+ cells as Eosinophils and SiglecF- cells. From SiglecF- cells we gated Ly6G+ neutrophils and Ly6G- cells. Ly6G- cells were used to gate CD11c+ Dendritic cells and F480+ Macrophages.


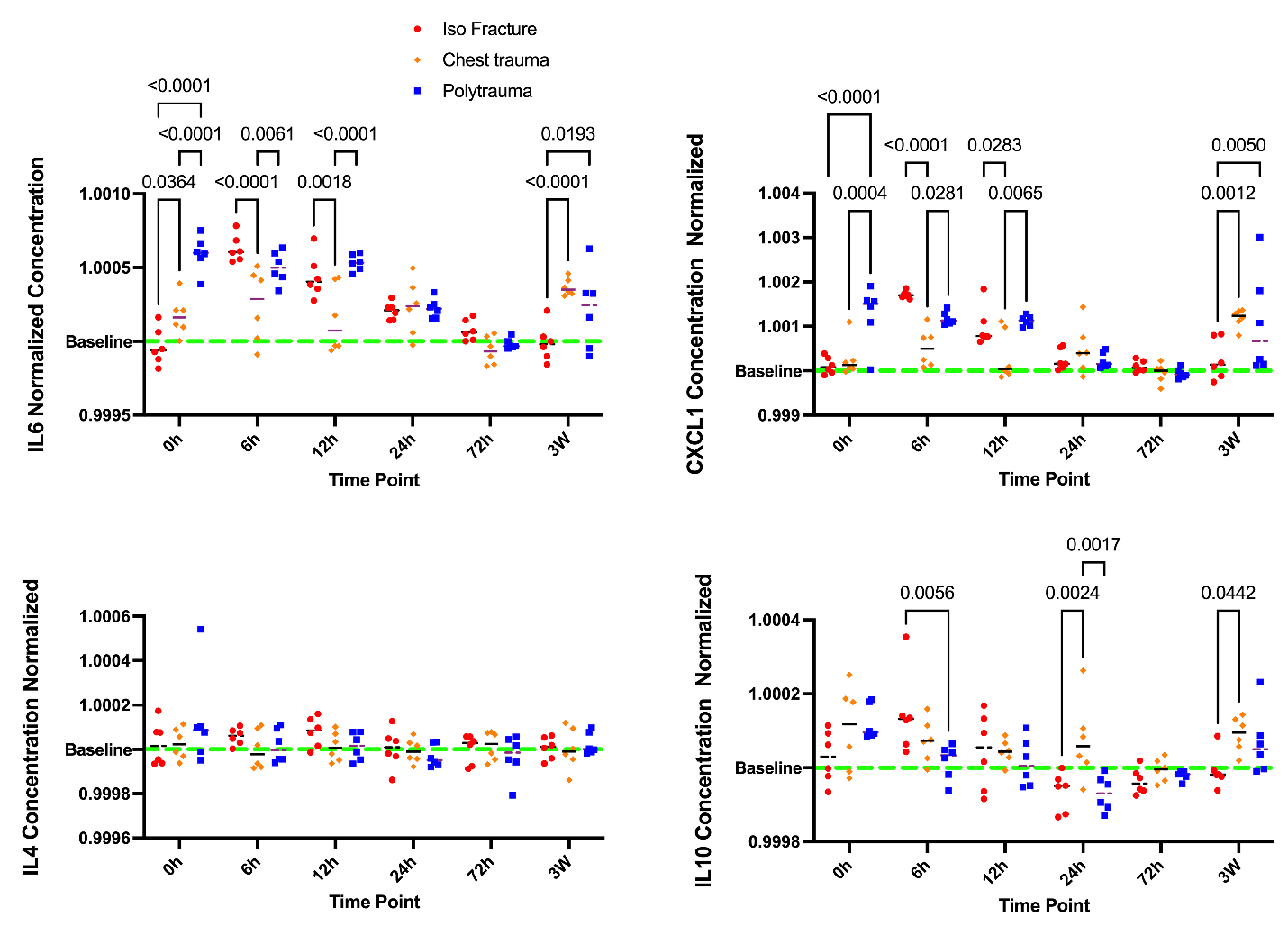


**Supplementary Figure 3.** Cytokine expression in polytrauma. The concentration of serum cytokines normalized to the healthy group (green line). N=6. Significant differences between groups were presented.


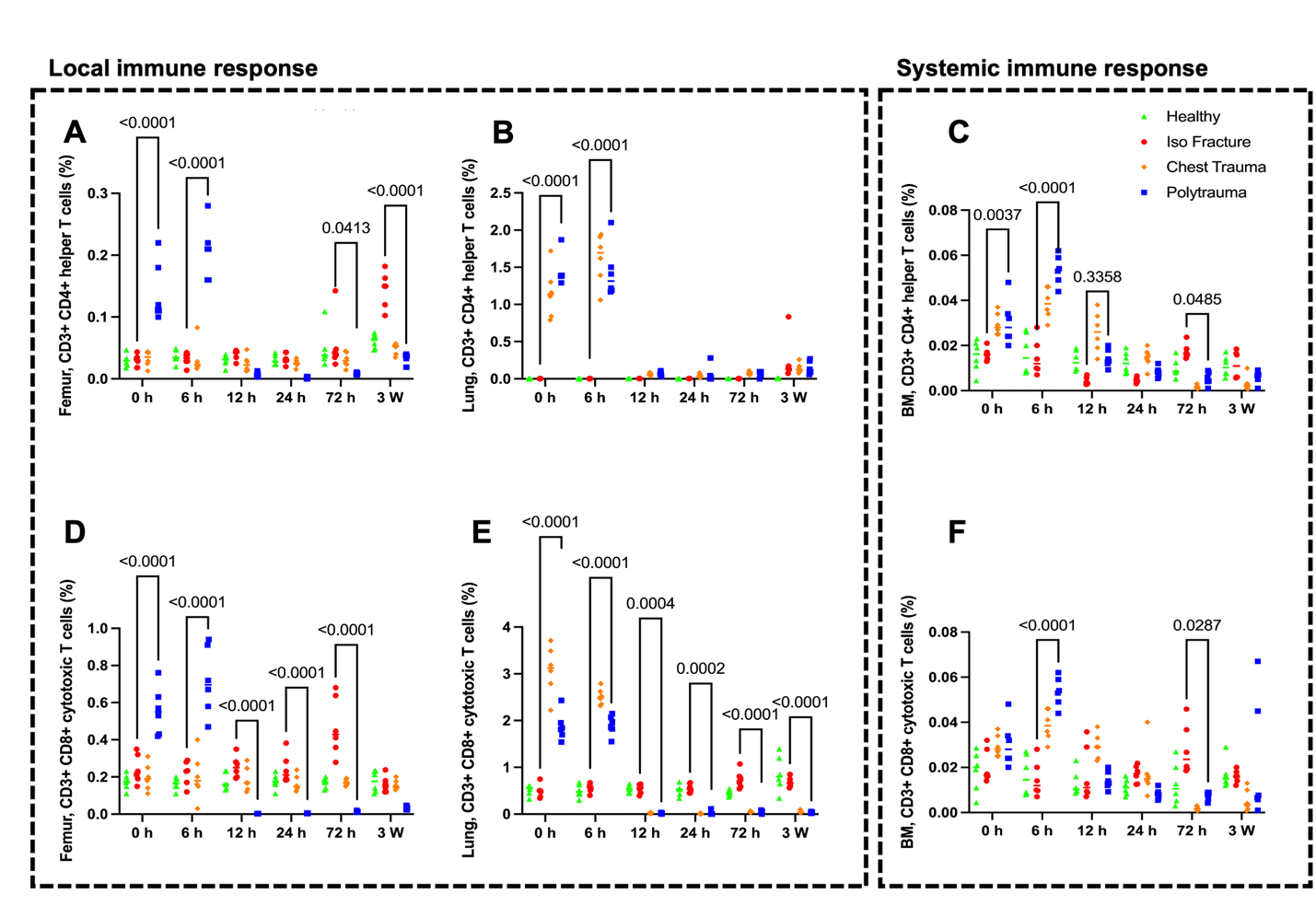


**Supplementary Figure 4.** Polytrauma induces significant differences in local and adaptive adaptive T cell responses to fracture healing. Flow cytometry data representing the percentage of CD3+CD4+ helper and CD3+CD8+ cytotoxic T cells at the femur fracture site (local, **A&D**), lungs (local, **B&E**), and bone marrow (systemic, **C&F**). BM (bone marrow). N=6. Significant differences between polytrauma and isolated fracture groups were presented. The percentages of CD3+CD4+ helper and CD3+CD8+ cytotoxic T cells were calculated relative to CD45^+^ cells.


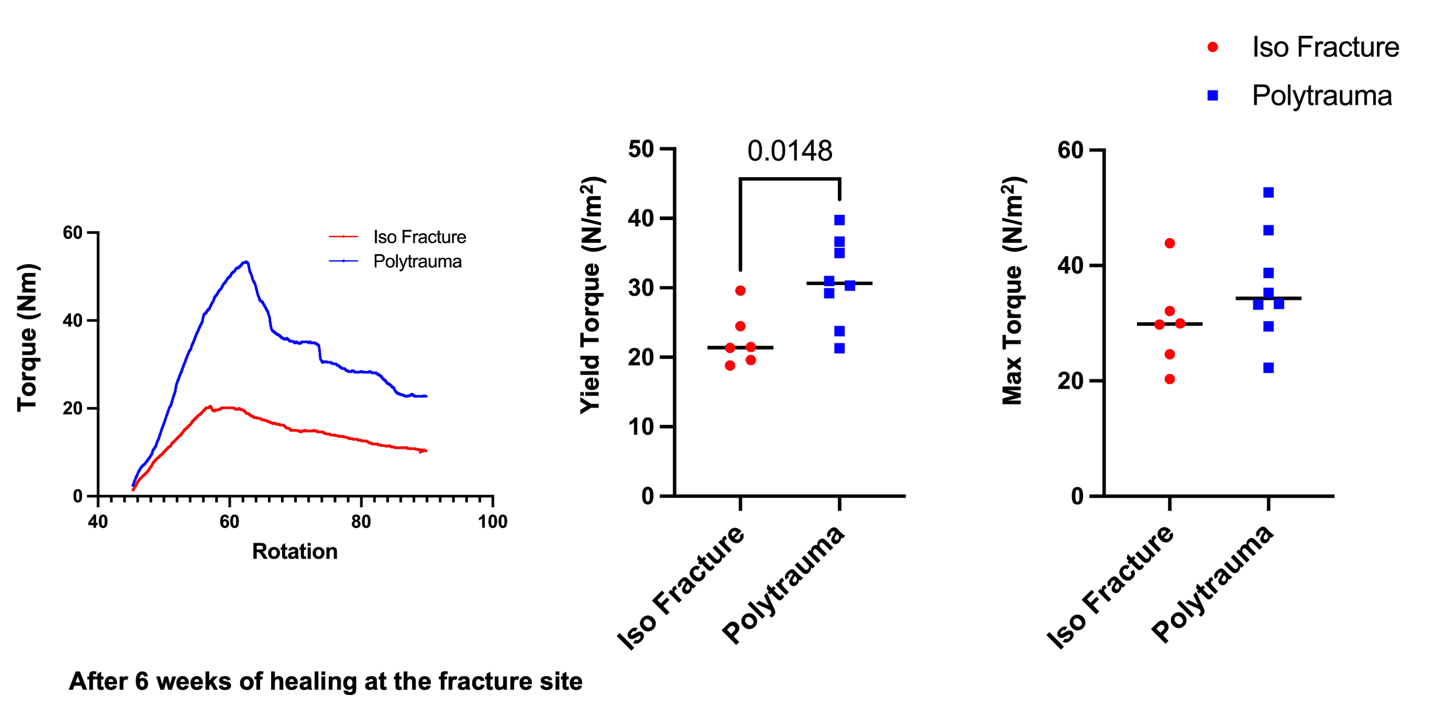


C

B

A

**Supplementary Figure 5.** Mechanical properties in polytrauma. **A-C**) Mechanical properties of callus from polytrauma and isolated fracture groups after 3 weeks of healing using torsion test. Compared to our original power analysis (N=8), the iso fracture (N=6) and polytrauma groups (N=8) experienced discrepancies in sample size due to sample loss during preparation or attrition from the study.


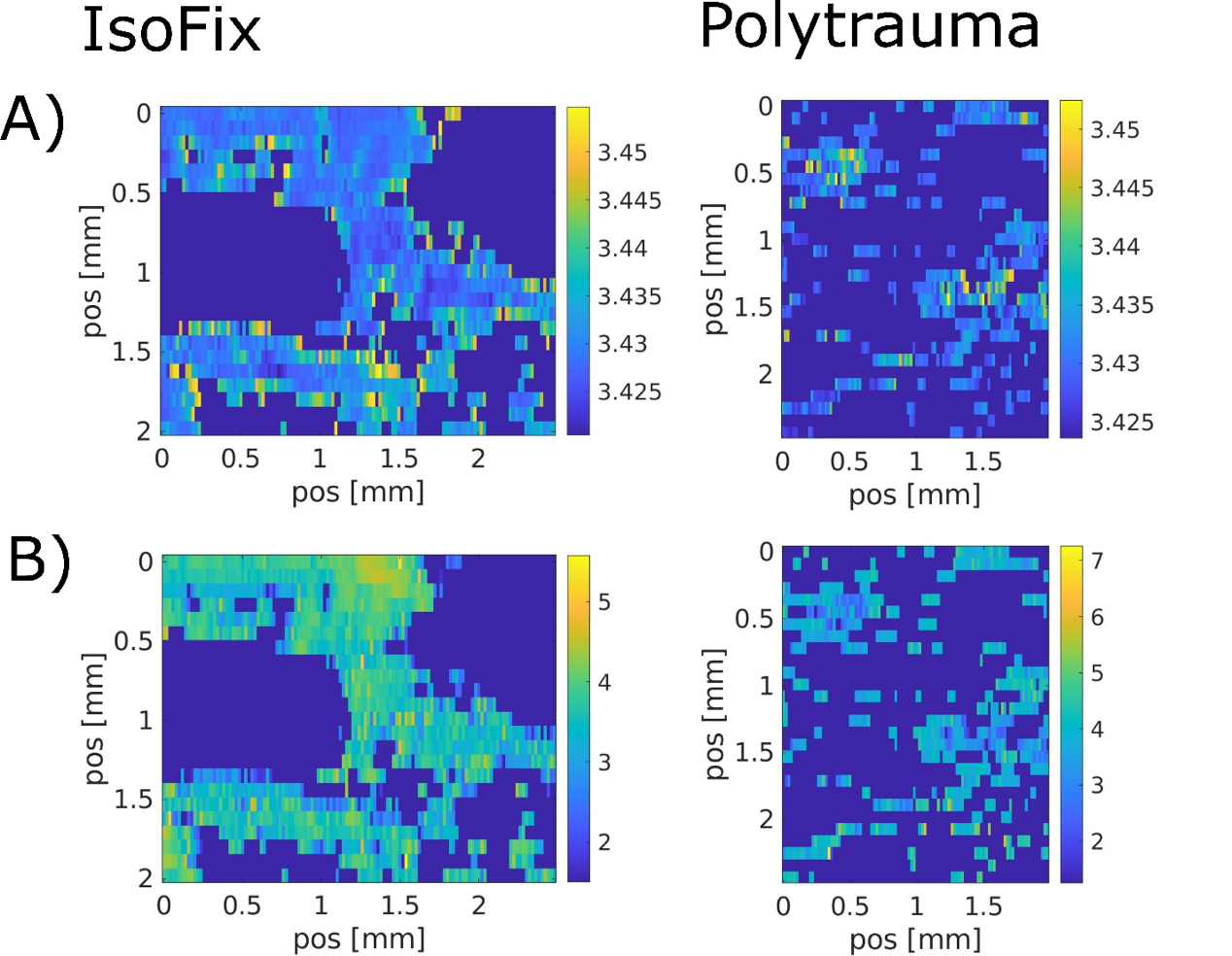


**Supplementary Figure 6.** Small angle X-ray scattering/X-ray diffraction (WAXS) analysis of the osteogenesis and [biomineralization](https://www.sciencedirect.com/topics/chemistry/biomineralization) processes at the fracture site in isolated fracture vs polytrauma groups. **A)** The lattice constant of the 002 reflection and **B)** calculated crystal size based on the full width of half maximum of the reflection in isolated fracture and polytrauma groups.


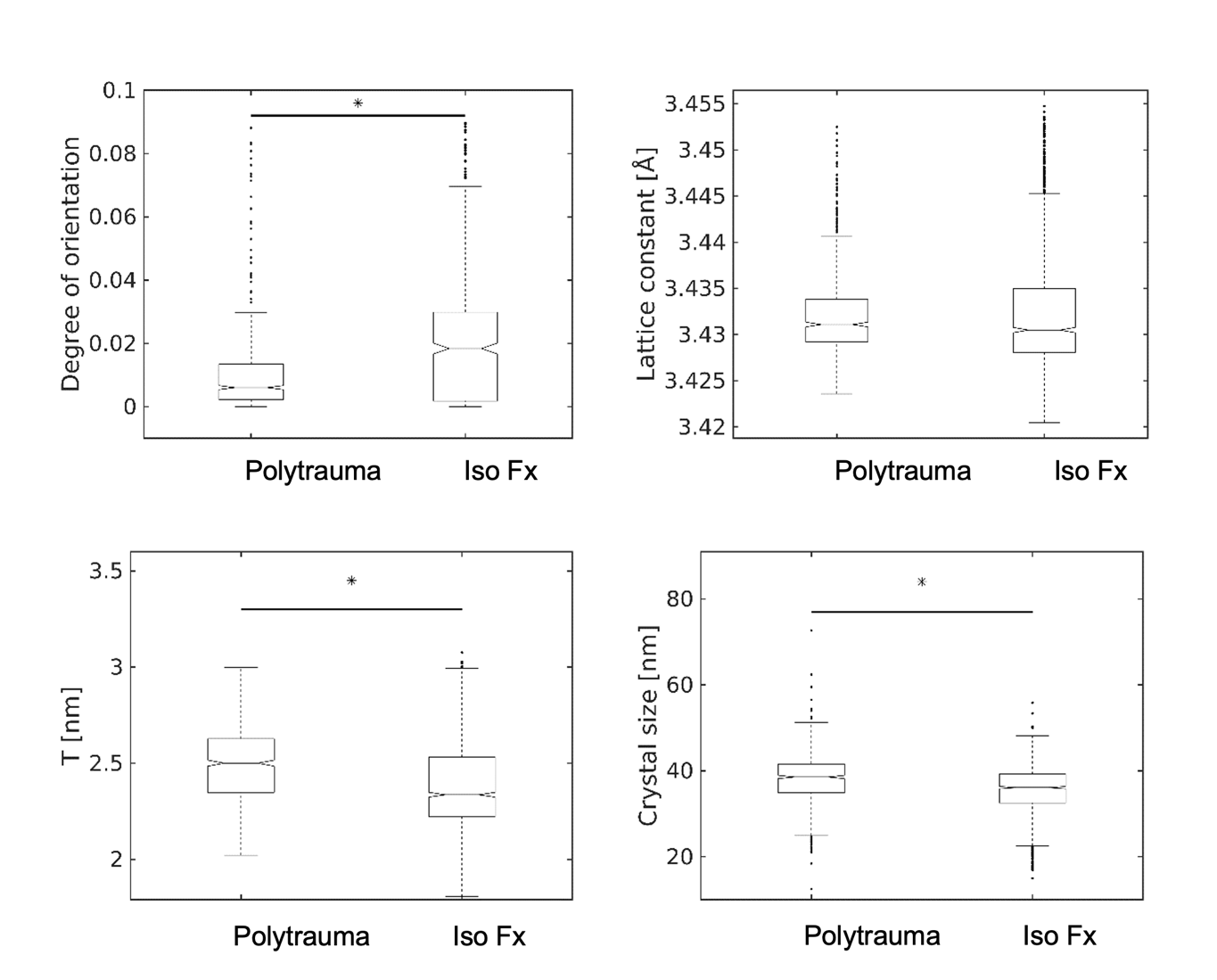


**Supplementary Figure 7.** Quantitative analysis of the extracted parameter from the scattering data. Degree of orientation, lattice constant of the 002 reflection, the mean crystal thickness (T parameter), and crystal size. Significant differences between polytrauma and isolated fracture groups are presented as *p< 0.05.
